# Supplementary material for: Quantitative mathematical modeling of clinical brain metastasis dynamics in non-small cell lung cancer
Source: Sci Rep. 2019 Sep 10;9:13018. doi: 10.1038/s41598-019-49407-3 (PMC6736889; doi:10.1038/s41598-019-49407-3)
Supplement: Supplementary file 1 — Supplementary Material [file 41598_2019_49407_MOESM1_ESM.pdf]

Quantitative mathematical modeling of clinical brain metastasis dynamics in non-small cell lung cancer

M. Bilous, C. Serdjebi, A. Boyer, P. Tomasini, C. Pouypoudat, D. Barbolosi, F. Barlesi, F. Chomy and S. Benzekry

Supplementary Material

Table S1: Growth kinetics of primary lung tumors

Figure S1: Comparison of exponential and Gompertz predictions of pre-clinical phase of growth

Figure S2: Gompertz growth predictions of individual brain metastases

Figure S3: Fit of the basic model

Figure S4: Fit of the model with secondary dissemination

Figure S5: Fit of the delay model

Figure S6: Fit of the model with different primary and secondary growth parameters

Figure S7: Fit of the dormancy model for patient 2

Figure S8: Predictions for patient 2

Figure S9: Shape of the objective as a function of μ and γ

Table S1: Growth kinetics of primary lung tumors

Ref.	All	Adenocarcinoma	Squamous cell carcinoma	Undiff.
[Jennings et al., 2006]	161 (149)	166 (51)	132 (48)	
[Usuda et al., 1994]	164 $\pm$ 178 (165)	223 $\pm$ 209 (86)	105 $\pm$ 106 (67)	79 $\pm$ 52 (12)
[Arai et al., 1994]	166 (237)	222 (133)	115 (69)	68 (9)
[Mizuno et al., 1984]	136 (50)	178 $\pm$ 157 (23)	103 $\pm$ 78 (22)	111 $\pm$ 57 (5)
[Geddes, 1979]	102 (228)	161 (60)	88 (111)	86 (44)
[Weiss, 1974]	183 $\pm$ 85 (28)	214 $\pm$ 73 (11)	78 $\pm$ 29 (8)	109 $\pm$ 72 (7)
[Spratt and Spratt, 1964]	88 (34)	118 (8)	70 (13)	93 (13)
[Spratt et al., 1963]	112 (22)	269 (7)	93 (6)	90 (9)
[Garland et al., 1963]	162 (41)	222 (7)	126 (22)	123 (9)
[Schwartz, 1961]	78 $\pm$ 48 (13)	72 $\pm$ 77 (2)	79 $\pm$ 46 (11)	
**Average***	**143**	**201**	**104**	**91**

Reports of volume doubling times from routinely detected primary NSCLC tumors from the literature (extended from [Detterbeck and Gibson, 2008] and [Friberg and Mattson, 1997]). Note: squamous cell carcinoma = epidermoid carcinoma and undifferentiated carcinoma cell carcinoma = large cell carcinoma. All denotes all histology from lung cancer together (including both NSCLC and small cell carcinomas).
Undiff. = undifferentiated carcinoma.
Mean $\pm$ standard deviation.
In parenthesis are the number of patients.
*Average was computed as the weighted mean of each study with weights the number of patients.

Figure S1: Comparison of exponential and Gompertz predictions of pre-clinical phase of growth

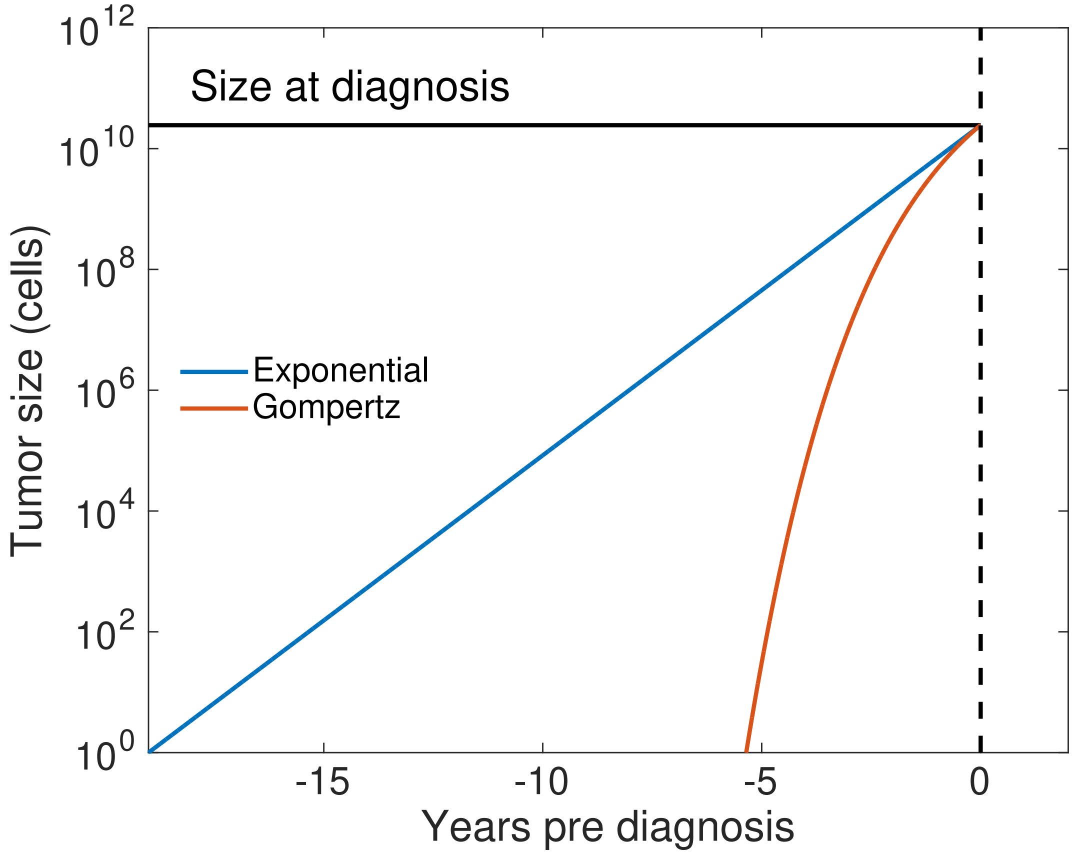

Figure S2: Gompertz growth predictions of individual brain metastases

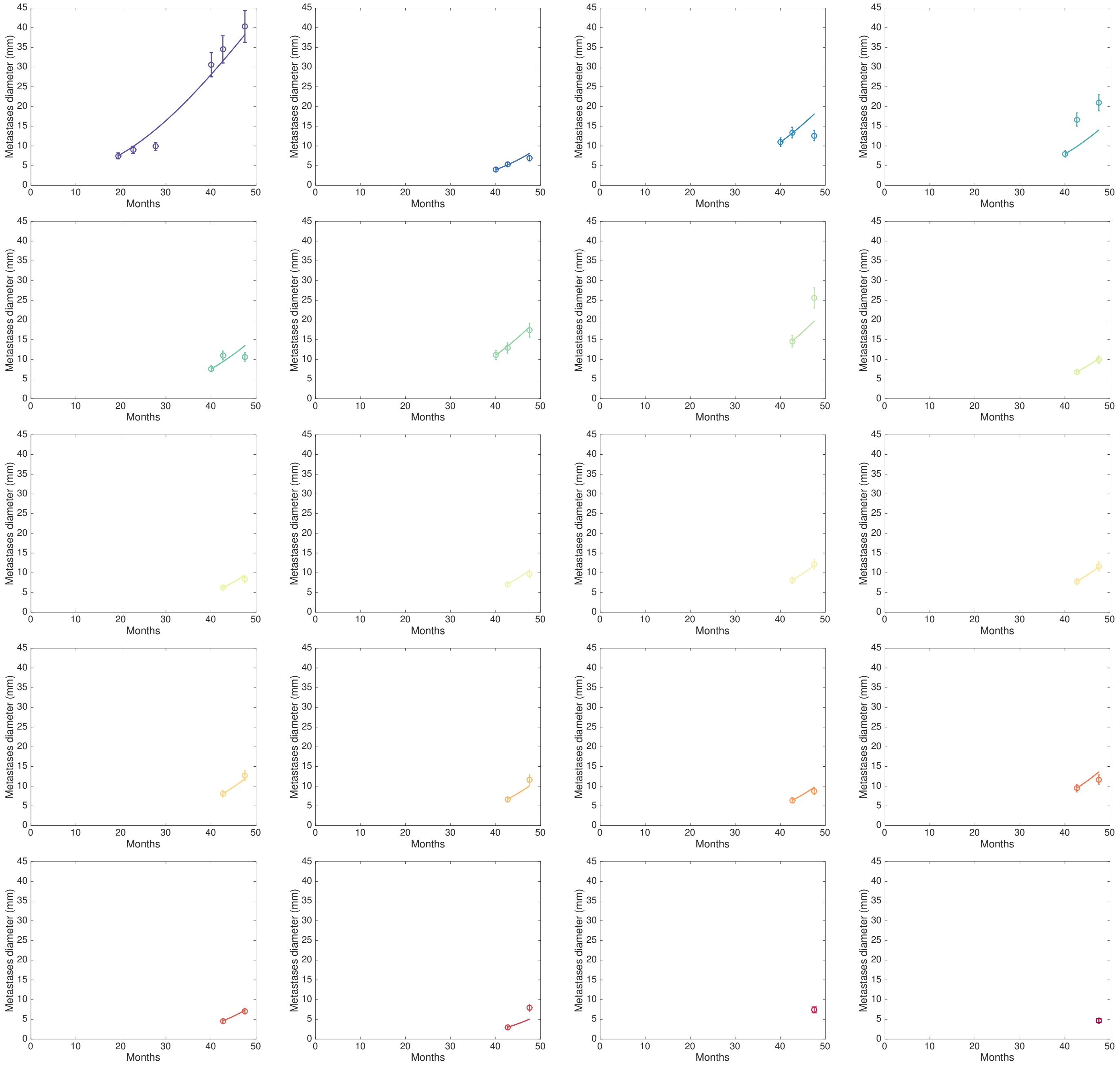

Comparison between the Gompertz growth model predictions and the lesion size data of individual BMs. The Gompertz growth parameters were determined only from the primary tumor size at diagnosis and histology (adenocarcinoma, see Methods). Apart from the first measurements used as initial conditions, no other future measurements were used to make the predictions. Error bars represent 10% error on the size measurement.

Figure S3: Fit of the basic model

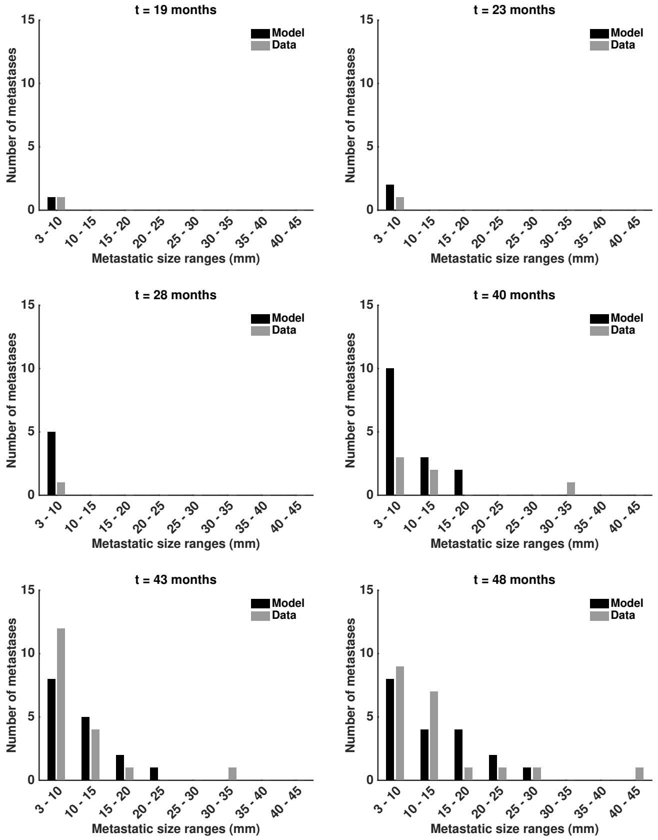

A

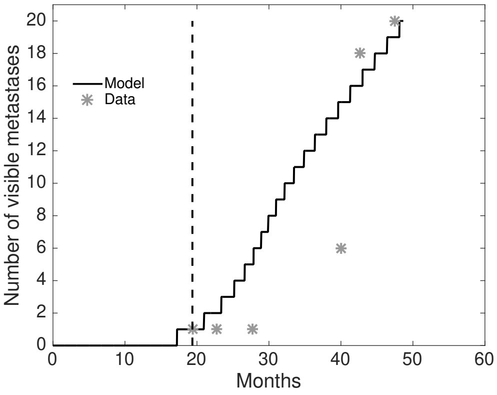

B

Parameter	Estimate	Unit	Method	Std. error
$\alpha_{0,p}$	0.0284	day^{-1}	Fixed	-
β_p	0.00103	day^{-1}	Fixed	-
α_0	0.0284	day^{-1}	Fixed	-
β	0.00103	day^{-1}	Fixed	-
μ	1.43×10^{-12}	cell$^{-\gamma}\times$ day^{-1}	Fit	3.33
γ	1	-	Variable	-
τ	0	day	Fixed	-
t_d	0	day	Fixed	-

C

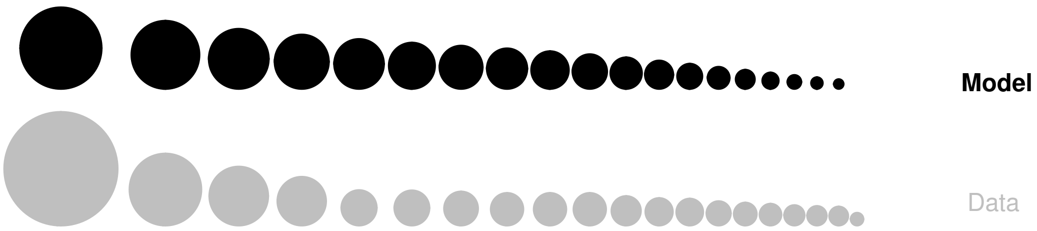

D

A. Time course of the visible brain metastases (BMs) size distributions during follow-up. Comparison between model calibration and data.
B. Time course of the number of visible BMs.
C. Parameter estimates. Std error = Standard errors expressed in percent.
D. Comparison of the BM size distribution between the model fit and the data at the last time point.

Figure S4: Fit of the model with secondary dissemination

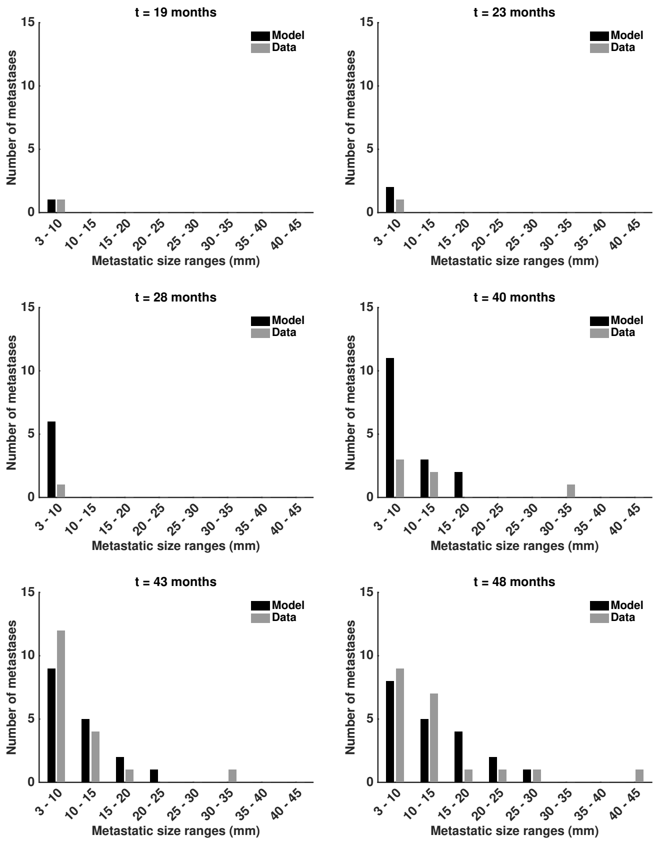

A

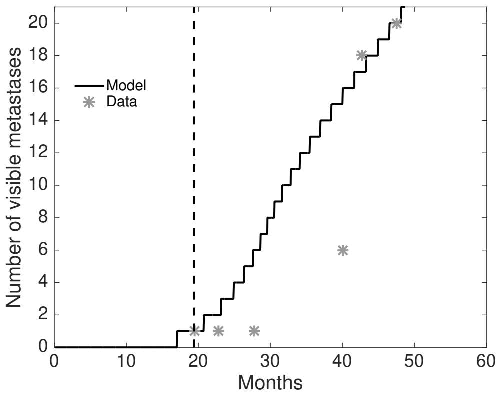

B

Parameter	Estimate	Unit	Method	Std. error
$\alpha_{0,p}$	0.0284	day^{-1}	Fixed	-
β_p	0.00103	day^{-1}	Fixed	-
α_0	0.0284	day^{-1}	Fixed	-
β	0.00103	day^{-1}	Fixed	-
μ	$1.5{\times}10^{-12}$	cell$^{-\gamma}\times$ day^{-1}	Fit	-
γ	1	-	Variable	-
τ	0	day	Fixed	-
t_d	0	day	Fixed	-

C

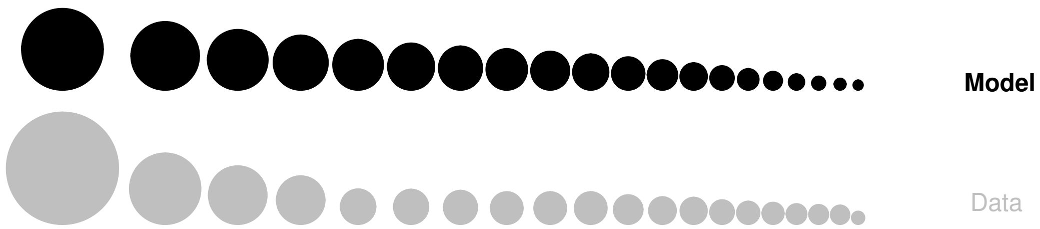

D

A. Time course of the visible brain metastases (BMs) size distributions during follow-up. Comparison between model calibration and data.

B. Time course of the number of visible BMs.

C. Parameter estimates. Std error = Standard errors expressed in percent.

D. Comparison of the BM size distribution between the model fit and the data at the last time point.

Figure S5: Fit of the delay model

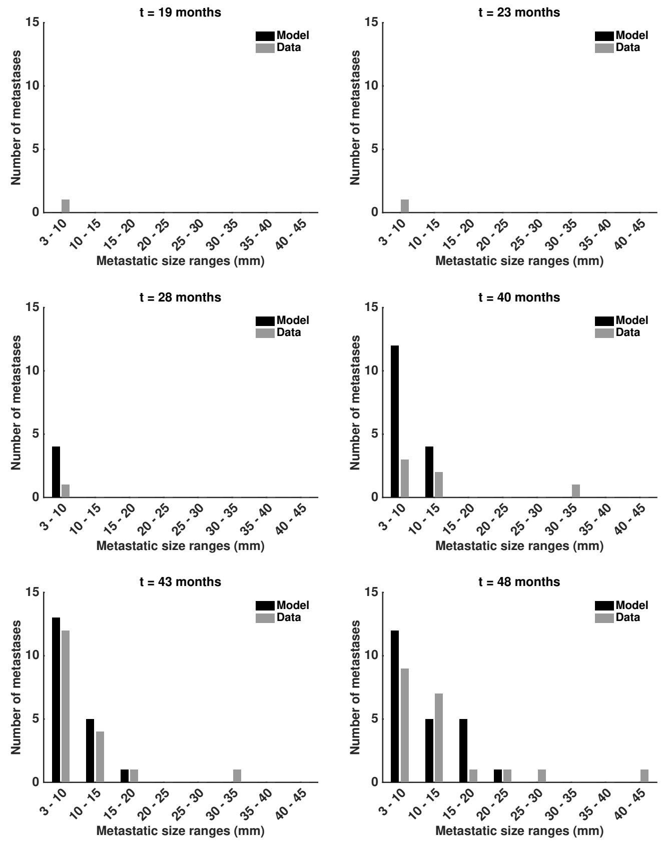

A

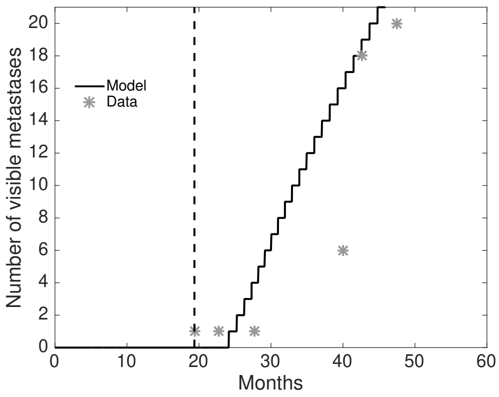

B

Parameter	Estimate	Unit	Method	Std. error
$\alpha_{0,p}$	0.0284	day^{-1}	Fixed	-
β_p	0.00103	day^{-1}	Fixed	-
α_0	0.0284	day^{-1}	Fixed	-
β	0.00103	day^{-1}	Fixed	-
μ	2.65×10^{-6}	cell$^{-\gamma}\times$ day^{-1}	Fit	10.5
γ	0.4	-	Variable	-
τ	0	day	Fixed	-
t_d	1.76×10^{3}	day	Fit	2.31

C

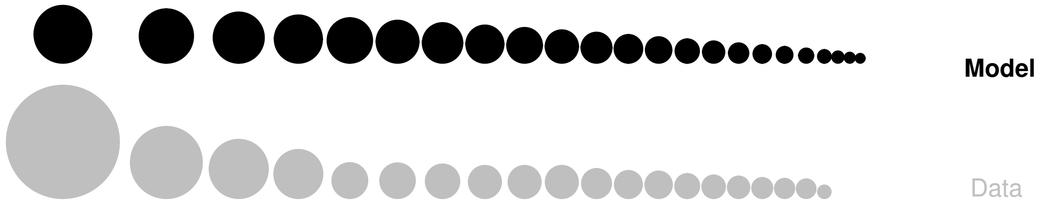

D

A. Time course of the visible brain metastases (BMs) size distributions during follow-up. Comparison between model calibration and data.

B. Time course of the number of visible BMs.

C. Parameter estimates. Std error = Standard errors expressed in percent.

D. Comparison of the BM size distribution between the model fit and the data at the last time point.

Figure S6: Fit of the model with different primary and secondary growth parameters

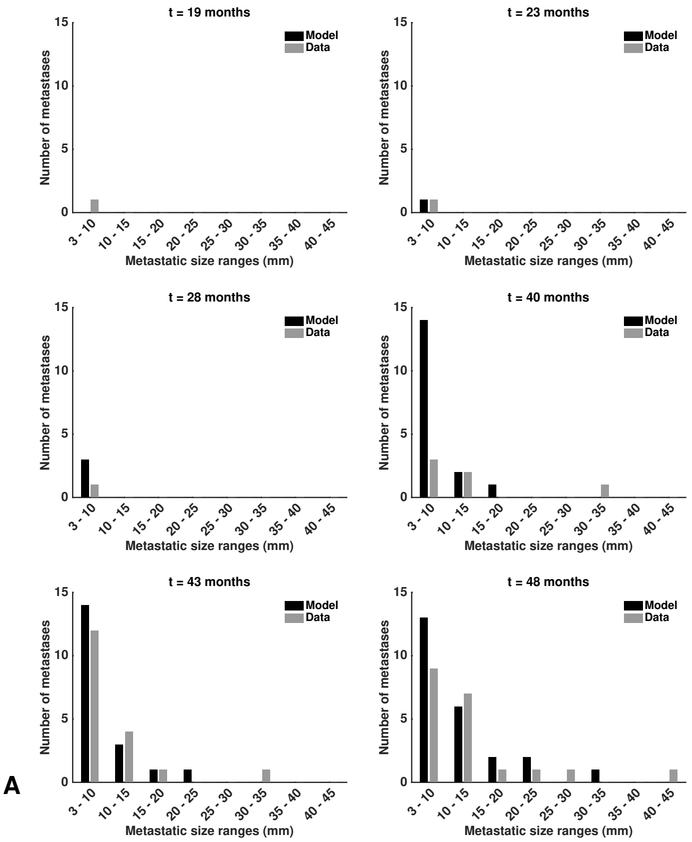

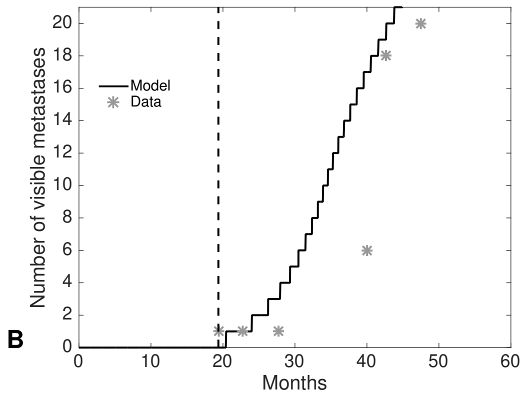

C

Parameter	Estimate	Unit	Method	Std. error
$\alpha_{0,p}$	0.0284	day^{-1}	Fixed	-
β_p	0.00103	day^{-1}	Fixed	-
α_0	0.022	day^{-1}	Fit	1.05
β	0.000666	day^{-1}	Fit	0.956
μ	2.07×10^{-12}	cell$^{-\gamma}\times$ day^{-1}	Fit	6.71
γ	1	-	Variable	-
τ	0	day	Fixed	-
t_d	0	day	Fixed	-

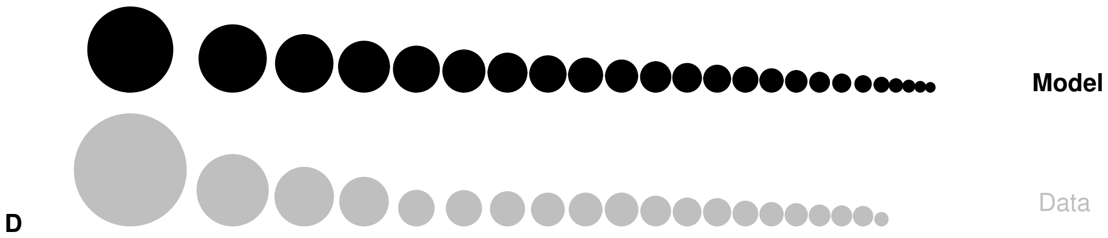

A. Time course of the visible brain metastases (BMs) size distributions during follow-up. Comparison between model calibration and data.

B. Time course of the number of visible BMs.

C. Parameter estimates. Std error = Standard errors expressed in percent.

D. Comparison of the BM size distribution between the model fit and the data at the last time point.

Figure S7: Fit of the dormancy model for patient 2

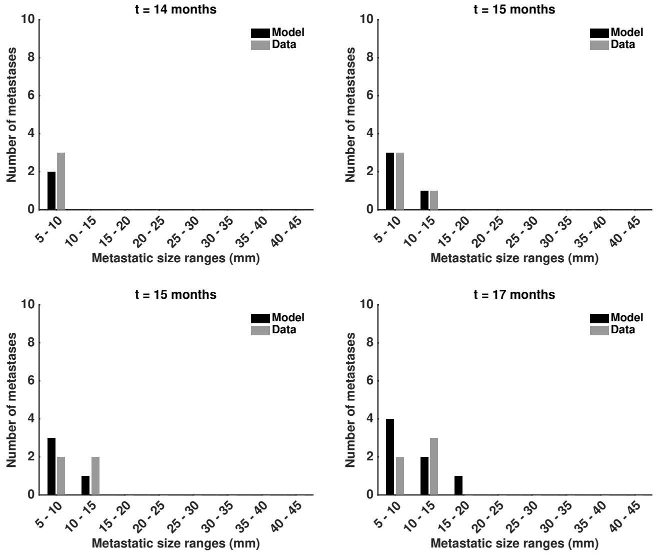

A

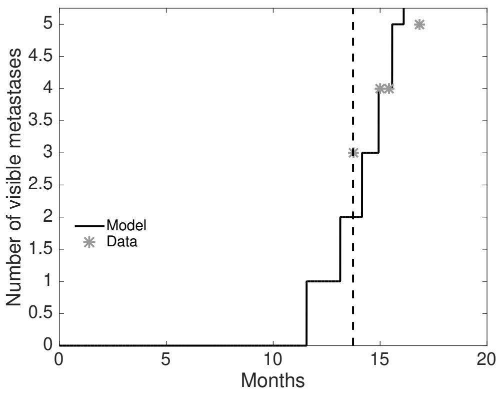

B

Parameter	Estimate	Unit	Method	Std. error
$\alpha_{0,p}$	0.0858	day^{-1}	Fixed	-
β_p	0.0031	day^{-1}	Fixed	-
α_0	0.0858	day^{-1}	Fixed	-
β	0.0031	day^{-1}	Fixed	-
μ	1.02×10^{-12}	cell$^{-\gamma}\times$ day^{-1}	Fit	71.9
γ	1	-	Variable	-
τ	171	day	Fit	33.3
t_d	0	day	Fixed	-

C

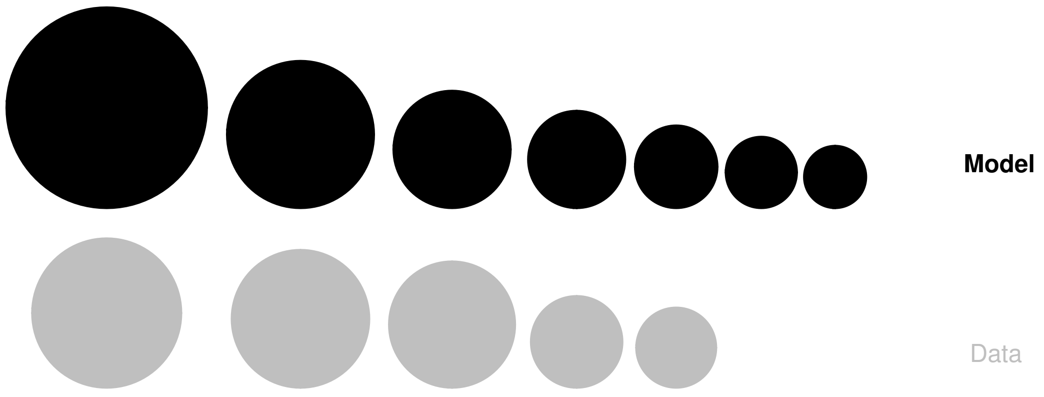

D

A. Time course of the visible brain metastases (BMs) size distributions during follow-up. Comparison between model calibration and data.

B. Time course of the number of visible BMs.

C. Parameter estimates. Std error = Standard errors expressed in percent.

D. Comparison of the BM size distribution between the model fit and the data at the last time point.

Figure S8: Predictions for patient 2

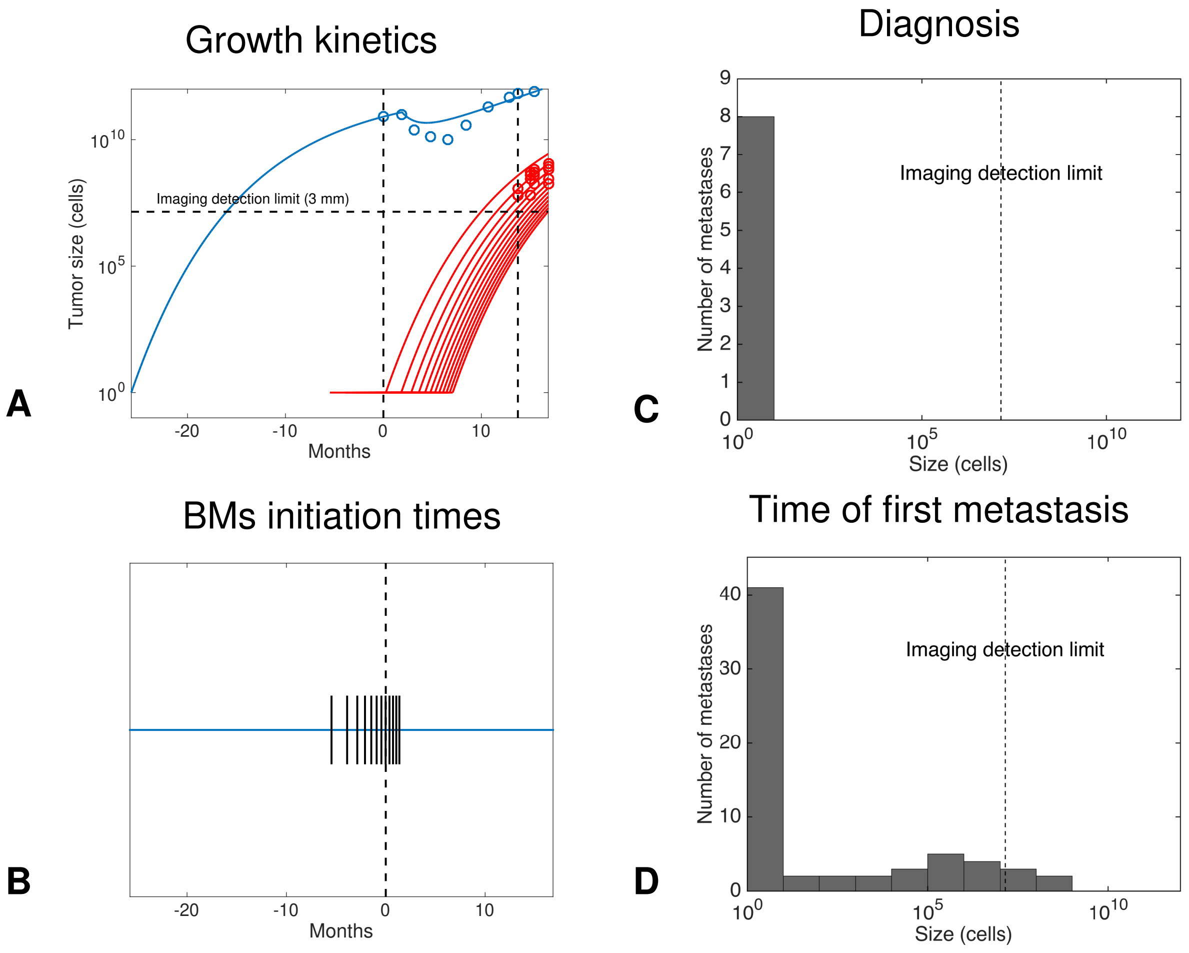

A. Inferred growth kinetics of the primary tumor (in blue) and the brain metastases (in red). Only brain metastases that will become visible are shown.

B. Model predictions of the initiation times of the brain metastases.

C. Predicted size distribution of the brain metastases at diagnosis.

D. Predicted size distribution of the brain metastases at the time of clinical occurrence of the first one.

Figure S9: Shape of the objective as a function of μ and γ

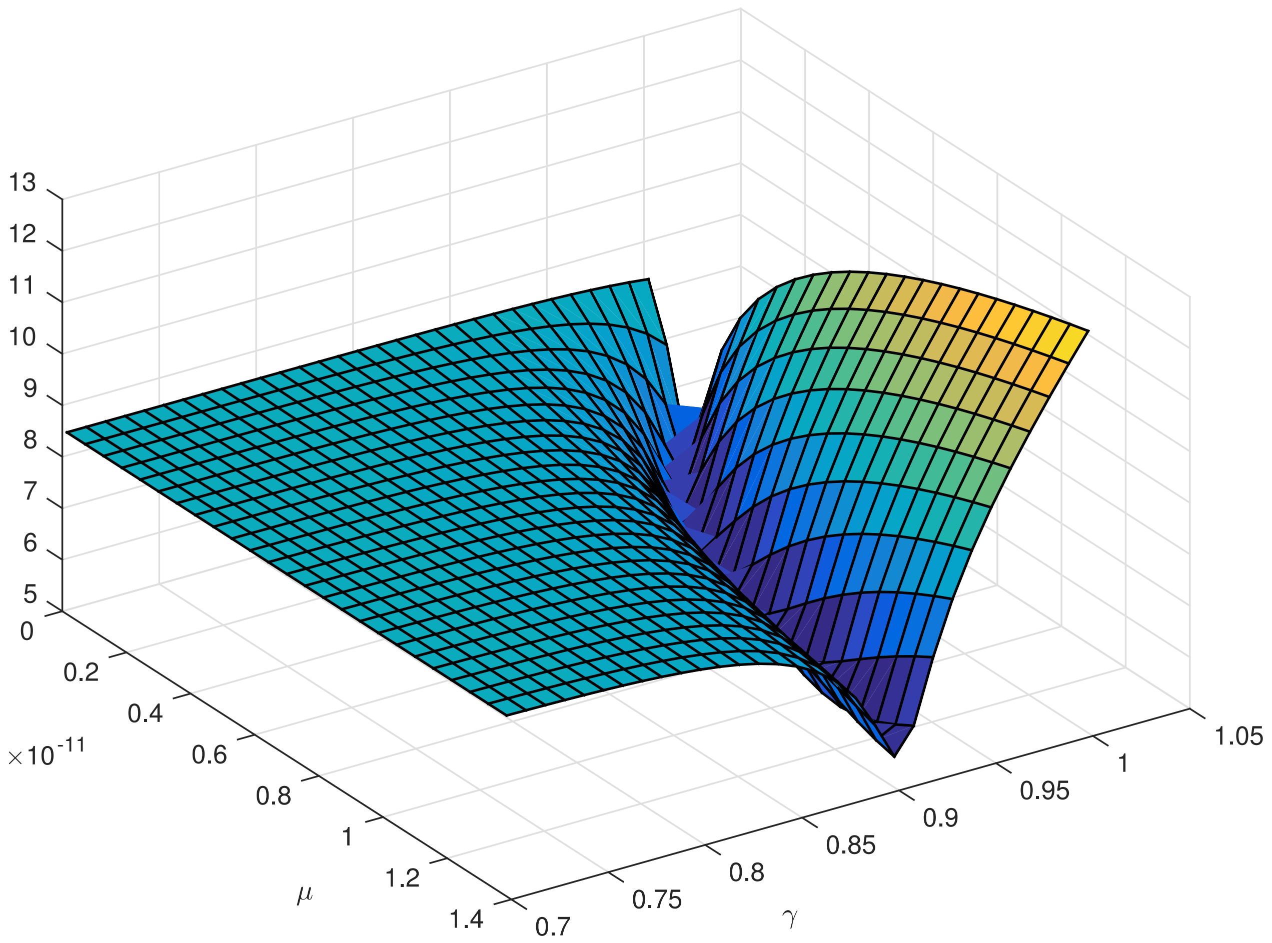

References

[Arai et al., 1994] Arai, T., Kuroishi, T., Saito, Y., Kurita, Y., Naruke, T., and Kaneko, M. (1994). Tumor doubling time and prognosis in lung cancer patients: evaluation from chest films and clinical follow-up study. Japanese Lung Cancer Screening Research Group. *Jpn J Clin Oncol*, 24(4):199–204.

[Detterbeck and Gibson, 2008] Detterbeck, F. C. and Gibson, C. J. (2008). Turning gray: the natural history of lung cancer over time. *J Thorac Oncol*, 3(7):781–792.

[Friberg and Mattson, 1997] Friberg, S. and Mattson, S. (1997). On the growth rates of human malignant tumors: implications for medical decision making. *J Surg Oncol*, 65(4):284–297.

[Garland et al., 1963] Garland, L. H., Coulson, W., and Wollin, E. (1963). The rate of growth and apparent duration of untreated primary bronchial carcinoma. *Cancer*, 16:694–707.

[Geddes, 1979] Geddes, D. M. (1979). The natural history of lung cancer: a review based on rates of tumour growth. *Br J Dis Chest*, 73(1):1–17.

[Jennings et al., 2006] Jennings, S. G., Winer-Muram, H. T., Tann, M., Ying, J., and Dowdeswell, I. (2006). Distribution of stage I lung cancer growth rates determined with serial volumetric CT measurements. *Radiology*, 241(2):554–563.

[Mizuno et al., 1984] Mizuno, T., Masaoka, A., Ichimura, H., Shibata, K., Tanaka, H., and Niwa, H. (1984). Comparison of actual survivorship after treatment with survivorship predicted by actual tumor-volume doubling time from tumor diameter at first observation. *Cancer*, 53(12):2716–2720.

[Schwartz, 1961] Schwartz, M. (1961). A biomathematical approach to clinical tumor growth. *Cancer*, 14:1272–1294.

[Spratt et al., 1963] Spratt, J. S., SPJUT, H. J., and ROPER, C. L. (1963). The frequency distribution of the rates of growth and the estimated duration of primary pulmonary carcinomas. *Cancer*, 16:687–693.

[Spratt and Spratt, 1964] Spratt, J. S. and Spratt, T. L. (1964). Rates of growth of pulmonray metastases and host survival. *Ann Surg*, 159(2):161–171.

[Usuda et al., 1994] Usuda, K., Saito, Y., Sagawa, M., Sato, M., Kanma, K., Takahashi, S., Endo, C., Chen, Y., Sakurada, A., and Fujimura, S. (1994). Tumor doubling time and prognostic assessment of patients with primary lung cancer. *Cancer*, 74(8):2239–2244.

[Weiss, 1974] Weiss, W. (1974). Tumor doubling time and survival of men with bronchogenic carcinoma. *Chest*, 65(1):3–8.
